# Low serum ficolin-3 levels are associated with severity and poor outcome in traumatic brain injury

**DOI:** 10.1186/s12974-015-0444-z

**Published:** 2015-12-01

**Authors:** Jian-Wei Pan, Xiong-Wei Gao, Hao Jiang, Ya-Feng Li, Feng Xiao, Ren-Ya Zhan

**Affiliations:** Department of Neurosurgery, The First Affiliated Hospital, School of Medicine, Zhejiang University, 79 Qingchun Road, Hangzhou, 310003 People’s Republic of China; Department of Neurosurgery, Sanmen People’s Hospital, 171 Renmin Road, Sanmen, 317100 People’s Republic of China

**Keywords:** Ficolin-3, Traumatic brain injury, Clinical outcome, Mortality

## Abstract

**Background:**

Ficolin-mediated activation of the lectin pathway of complement contributes to the complement-independent inflammatory processes of traumatic brain injury. Lower serum ficolin-3 levels have been demonstrated to be highly associated with unfavorable outcome after ischemic stroke. This prospective observatory study was designed to investigate the relationships between serum ficolin-3 levels and injury severity and clinical outcomes after severe traumatic brain injury.

**Methods:**

Serum ficolin-3 levels of 128 patients and 128 healthy controls were measured by sandwich immunoassays. An unfavorable outcome was defined as Glasgow Outcome Scale score of 1–3. Study endpoints included mortality at 1 week and 6 months and unfavorable outcome at 6 months after head trauma. Injury severity was assessed by Glasgow Coma Scale score. Multivariate logistic models were structured to evaluate the relationships between serum ficolin-3 levels and study endpoints and injury severity.

**Results:**

Compared with the healthy controls, serum ficolin-3 levels on admission were statistically decreased in patients with severe traumatic brain injury. Serum ficolin-3 levels were independently correlated with Glasgow Coma Scale scores. Ficolin-3 was also identified as an independent prognostic predictor for 1-week mortality, 6-month mortality, and 6-month unfavorable outcome. Under receiver operating characteristics curves, ficolin-3 has similar prognostic predictive values for all study endpoints compared with Glasgow Coma Scale scores.

**Conclusions:**

It was proposed that lower serum ficolin-3 levels, correlated with injury severity, had the potential to be the useful, complementary tool to predict short- or long-term clinical outcomes after severe traumatic brain injury.

## Background

Severe traumatic brain injury (STBI) is a serious public health problem [1–3]. Outcome prediction is relevant for both clinical practice and research of STBI [4–6]. Low Glasgow Coma Scale (GCS) scores are associated highly with poor clinical outcomes of STBI patients [7–9]. In recent years, the application of biomarkers identified in the peripheral blood has shown potential clinical utility in neurointensive care as diagnostic, prognostic, and monitoring adjuncts [10–12]. Identifying sensitive and reliable biomarkers associated with patient outcome may improve our understanding of structural brain damage or underlying cellular pathogenesis and regenerative mechanisms after brain trauma. This information can be used to guide future basic and clinical research, therefore improving patient care and outcomes [13, 14].

The complement system is an integral part of the innate immune system and inflammation [15–17]. Three distinct pathways constitute the complement system: the classical pathway, the alternative pathway, and the lectin pathway [18]. The C1 complex initiates the classical pathway upon recognition of immune complexes and dying host cells [19]. The alternative pathway is spontaneously activated by C3 hydrolysis, but it has also been reported that properdin, a stabilizer of the alternative pathway convertase [20], is capable of initiating the complement cascade [21]. The ficolins and mannose-binding lectin (MBL) in association with MBL/ficolin-associated serine proteases (MASPs) are the initiator molecules of the lectin pathway. Three MASPs (−1, −2, and −3) have been described so far, and the current notion is that MASP-2 is the main lectin pathway activator. Upon recognition of pathogen-associated molecular patterns or altered self by MBL and the ficolins, the associated proteases cleave C4 and C2, hereby activating the complement cascade which ultimately leads to the formation of the terminal complement complex [22]. Various studies have revealed particularly novel findings on the wide-ranging involvement of complement in neural development, synapse elimination, and maturation of neural networks, as well as the progression of pathology in a range of acute and chronic neurodegenerative disorders [23–26].

A traumatic impact to the brain induces an intracranial inflammatory response, which consequently leads to the development of brain edema and delayed neuronal death [27–29]. Evidence from experimental, clinical, and in vitro studies highlights an important role for ficolin-mediated activation of the lectin pathway of complement in contributing to inflammation within the injured brain [30–32]. To date, three different ficolins (−1, −2, and −3) derived respectively from the genes FCN1, FCN2, and FCN3 have been described in humans [33]. Ficolin-3 was firstly identified as a serum glycoprotein that reacted with autoantibodies from patients with systemic lupus erythematosus [34]. Ficolin-3 is proved to be the predominant plasma molecule and the greatest complement-activating capacity among the lectin pathway initiators [35–40]. Thus, it may possess the strongest potential to be a prognostic or diagnostic biomarker. Recently, lower serum ficolin-3 levels have been demonstrated to be highly associated with the severity and unfavorable outcome after acute ischemic stroke [41]. Moreover, low levels of plasma ficolin-3 were related to severity, vasospasm, and cerebral ischemia of aneurysmal subarachnoid hemorrhage [42]. Hence, we further investigated the ability of ficolin-3 to predict short- and long-term clinical outcomes of STBI patients.

## Methods

### Study populations

This is a prospective observatory study conducted during the period of 3 years from July 2011 to July 2014 at the Sanmen People’s Hospital, Zhejiang Province, China. This study included the patients with isolated head trauma and postresuscitation GCS score of 8 or less. Exclusion criteria were infectious diseases; fever within recent 1 month before head trauma; an elevated white blood cell count; positive chest X-ray, less than 18 years of age; admission time >6 h; previous head trauma; neurological disease including ischemic or hemorrhagic stroke; use of antiplatelet or anticoagulant medication; and the presence of other prior systemic diseases including diabetes mellitus, hypertension, uremia, liver cirrhosis, malignancy, and chronic heart or lung disease.

Control subjects were recruited from volunteers who attended the Sanmen People’s Hospital for healthy examination between July 2011 and July 2014. They showed normal blood and biochemical laboratory tests, namely differential blood count, hemoglobin level, total serum proteins, liver function tests, erythrocytes sedimentation rate, kidney function tests, and C-reactive proteins as well as were medically tested by a specialist and found free of any other medical illness.

The study was approved by the Human Investigations Committee at the Sanmen People’s Hospital, and written informed consent was obtained from the healthy controls and the legal guardians of STBI patients. This study was registered in ClinicalTrials.gov (NCT02510573) on 22 July 2015 by Xiong-Wei Gao.

### Assessment

Head trauma severity was assessed using initial postresuscitation GCS score. All computerized tomography (CT) scans were performed according to the neuroradiology department protocol. Investigators who read them were blinded to clinical information. Abnormal cisterns, midline shift >5 mm, and subarachnoid hemorrhage (SAH) were recorded on initial CT scan. CT classification was performed using Traumatic Coma Data Bank criteria on initial postresuscitation CT scan according to the method of Marshall et al. Participants were followed up until death or completion of 6 months after head trauma. Clinical outcome included 1-week mortality, 6-month mortality, and 6-month functional outcome. Using structure telephone interviews, follow-up was performed by one doctor who was blinded to clinical information.

### Immunoassay methods

Venous blood was drawn from patients on admission and from healthy controls at study entry. Serum was frozen at −70 °C until assayed. Serum ficolin-3 concentration was determined by enzyme-linked immunosorbent assay (Hycult Biotech, the Netherlands). The intra-assay and inter-assay variations were 3.8 and 5.9 %, respectively. All determinations were run in duplicates. The persons carrying out the assays were completely blinded to the clinical information.

### Statistical analysis

The results were reported as counts (percentage) for the categorical variables, mean ± standard deviation if normally distributed, and median (interquartile range) if not normally distributed for the continuous variables. Comparisons were made by using (1) chi-square test or Fisher’s exact test for categorical data, (2) Student’s *t* test for continuous normally distributed variables, and (3) the Mann–Whitney *U* test for continuous non-normally distributed variables. Correlations were analyzed by Spearman’s correlation coefficient or Pearson’s correlation coefficient and then followed by a multivariate linear regression.

The relations of ficolin-3 levels to clinical outcomes were assessed in a logistic-regression model with calculated odds ratio (OR) and 95 % confidence interval (CI). For multivariate analysis, we included the significantly different outcome predictors as assessed in univariate analysis. Under receiver operating characteristic (ROC) curve, the area under curve (AUC) was calculated to assess the predictive performance of ficolin-3 levels for clinical outcomes. A combined logistic-regression model was configured to estimate the additive benefit of ficolin-3 levels to GCS scores.

Statistical analysis was performed with SPSS 19.0 (SPSS Inc., Chicago, IL, USA) and MedCalc 9.6.4.0. (MedCalc Software, Mariakerke, Belgium). A *P* value of less than 0.05 was considered statistically significant.

## Results

### Study populations’ characteristics

During the study period, 164 patients were admitted to our emergency department with an isolated severe head trauma diagnosis. Of these, 36 patients were excluded because of the following reasons. Five cases had neurological diseases; five cases had infectious diseases; two cases, fever within recent 1 month; four cases, elevated white blood cell count; four cases, admission >6 h; four cases, the presence of other systemic diseases; two cases, previous head trauma; three cases, positive chest X-ray; two cases, less than 18 years of age; two cases, missing of follow-up; and four cases, use of antiplatelet or anticoagulant medication. Finally, 128 patients were included in the analysis.

This group of patients, consisting of 80 men and 48 women, had a mean age of 42.5 ± 15.6 years. Median initial postresuscitation GCS scores were 5 (3). Sixty-two patients (48.4 %) had unreactive pupils on admission; 58 patients (45.3 %), CT classification 5 or 6; 60 patients (46.9 %), abnormal cisterns on initial CT scan; 65 patients (50.8 %), midline shift >5 mm on initial CT scan; 70 patients (54.7 %), the presence of traumatic subarachnoid hemorrhage on initial CT scan; and 58 patients (45.3 %), intracranial surgery in the first 24 h. The mean admission time was 2.5 ± 1.3 h; the mean plasma-sampling time, 3.8 ± 1.6 h; the mean systolic arterial pressure, 129.9 ± 27.5 mmHg; the mean diastolic arterial pressure, 77.5 ± 17.9 mmHg; the mean value of mean arterial pressure 96.5 ± 18.4 mmHg; the mean plasma C-reactive protein levels, 7.9 ± 3.2 mg/L; and the mean blood glucose levels, 11.1 ± 3.9 mmol/L.

Control group, consisting of 128 healthy individuals, included 83 men and 45 women and had a mean age of 42.2 ± 16.4 years. Differences in gender and age were not shown to be statistically significant between control group and patients.

### Change of serum ficolin-3 levels

**Fig. 1:**
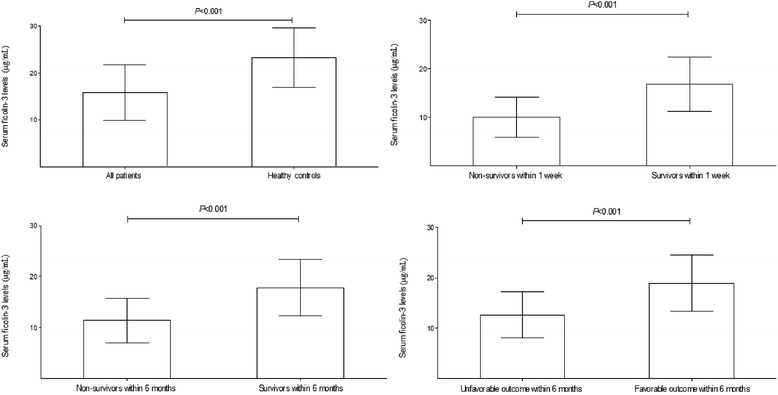
Graph showing change of serum ficolin-3 levels after severe traumatic brain injury

Just as shown in Fig. 1, the admission serum ficolin-3 levels were significantly lower in all patients than in healthy controls, in patients dying than in patients alive within 1 week, in patients dying than in patients alive within 6 months, and in patients with unfavorable outcome than in patients with favorable outcome within 6 months.

### Correlation analysis

**Fig. 2:**
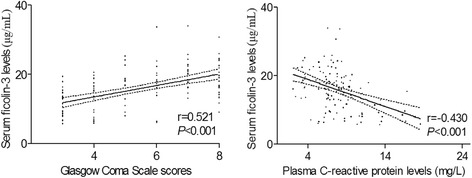
Graph showing the relationships between serum ficolin-3 levels and Glasgow Coma Scale scores as well as between serum ficolin-3 levels and plasma C-reactive protein levels after severe traumatic brain injury

Just as shown in Table 1, serum ficolin-3 levels were highly associated with initial postresuscitation GCS scores, pupils unreactive on admission, CT classification 5 or 6, abnormal cisterns on initial CT scan, midline shift >5 mm on initial CT scan, the presence of traumatic SAH on initial CT scan, intracranial surgery in the first 24-h blood glucose level, and plasma C-reactive protein levels. A multivariate linear regression demonstrated that serum ficolin-3 levels were still highly associated with GCS scores (*t* = 4.994, *P* < 0.001) and plasma C-reactive protein levels (*t* = −3.005, *P* = 0.003) in Fig. 2.

**Table 1:** The factors correlated with serum ficolin-3 levels in patients with severe traumatic brain injury

Characteristics	*r* value	*P* value
Sex (male/female)	0.092	0.303
Age (years)	0.046	0.606
Initial postresuscitation GCS score	0.521	<0.001
Pupils unreactive on admission	−0.414	<0.001
CT classification 5 or 6	−0.189	0.033
Abnormal cisterns on initial CT scan	−0.300	0.001
Midline shift >5 mm on initial CT scan	−0.323	<0.001
Presence of traumatic SAH on initial CT scan	−0.303	0.001
Intracranial surgery in the first 24 h	−0.245	0.005
Admission time (h)	0.090	0.310
Plasma-sampling time (h)	0.067	0.450
Systolic arterial pressure (mmHg)	0.127	0.152
Diastolic arterial pressure (mmHg)	0.120	0.179
Mean arterial pressure (mmHg)	0.132	0.137
Plasma C-reactive protein levels (mg/L)	−0.430	<0.001
Blood glucose levels (mmol/L)	−0.285	<0.001

Bivariate correlations were assessed by Spearman’s or Pearson’s correlation coefficient

*GCS* Glasgow Coma Scale, *CT* computerized tomography, *SAH* subarachnoid hemorrhage

### Prediction of 1-week mortality

In Table 2, serum ficolin-3 levels and other variables were highly associated with 1-week mortality of patients with STBI. A multivariate analysis demonstrated that GCS scores (OR 0.298, 95 % CI 0.138–0.646, *P* < 0.001) and serum ficolin-3 levels (OR 0.821, 95 % CI 0.704–0.958, *P* < 0.001) were the independent predictors for 1-week mortality of patients.

**Table 2:** The factors associated with poor clinical outcomes in patients with severe traumatic brain injury

Characteristics	1-week mortality prediction			6-month mortality prediction			6-month functional outcome prediction		
	Non-survivors (*n* = 20)	Survivors (*n* = 108)	*P* value	Non-survivors (*n* = 40)	Survivors(*n* = 88)	*P* value	Unfavorable outcome (*n* = 64)	Favorable outcome (*n* = 64)	*P* value
Sex (male/female)	13/7	67/41	0.801	27/13	53/35	0.431	44/20	36/28	0.144
Age (years)	45.0 ± 14.5	42.1 ± 15.8	0.440	43.8 ± 15.5	41.9 ± 15.7	0.522	44.6 ± 16.0	40.4 ± 15.1	0.132
Initial postresuscitation GCS score	3 (1)	6 (4)	<0.001	3 (1)	6 (3)	<0.001	4 (2)	7 (3)	<0.001
Pupils unreactive on admission	18 (90.0 %)	44(40.7 %)	<0.001	32 (80.0 %)	30 (34.1 %)	<0.001	43 (80.0 %)	19 (34.1 %)	<0.001
CT classification 5 or 6	15 (75.0 %)	43 (39.8 %)	0.004	25 (62.5 %)	33 (37.5 %)	0.008	36 (56.3 %)	22 (34.4 %)	0.013
Abnormal cisterns on initial CT scan	18 (90.0 %)	42(38.9 %)	<0.001	29 (72.5 %)	31(35.2 %)	<0.001	40 (62.5 %)	20(31.3 %)	<0.001
Midline shift >5 mm on initial CT scan	17 (85.0 %)	48 (44.4 %)	0.001	30 (75.0 %)	35(39.8 %)	<0.001	42 (65.6 %)	23(35.9 %)	0.001
Presence of traumatic SAH on initial CT scan	16 (80.0 %)	54(50.0 %)	0.013	29 (72.5 %)	41(46.6 %)	0.006	41 (64.1 %)	29 (45.3 %)	0.033
Intracranial surgery in the first 24 h	12 (60.0 %)	46 (42.6 %)	0.151	21 (52.2 %)	37 (42.1 %)	0.271	31 (48.4 %)	27(42.2 %)	0.478
Admission time (h)	2.3 ± 0.9	2.5 ± 1.3	0.446	2.3 ± 0.9	2.5 ± 1.4	0.384	2.3 ± 1.1	2.6 ± 1.4	0.125
Plasma-sampling time (h)	4.2 ± 1.2	3.7 ± 1.6	0.183	3.7 ± 1.2	3.8 ± 1.7	0.627	3.6 ± 1.5	3.9 ± 1.6	0.220
Systolic arterial pressure (mmHg)	139.1 ± 22.6	128.2 ± 28.0	0.105	126.7 ± 30.7	131.4 ± 25.9	0.370	126.4 ± 31.2	133.5 ± 22.9	0.146
Diastolic arterial pressure (mmHg)	81.7 ± 12.6	76.7 ± 18.7	0.255	77.6 ± 16.5	77.4 ± 18.6	0.942	75.5 ± 20.0	79.4 ± 15.5	0.219
Mean arterial pressure (mmHg)	102.7 ± 15.1	95.4 ± 18.8	0.100	99.0 ± 16.5	95.4 ± 19.2	0.311	95.6 ± 20.4	97.4 ± 16.3	0.572
Plasma C-reactive protein level (mg/L)	11.1 ± 2.3	7.3 ± 3.1	<0.001	10.3 ± 4.1	6.8 ± 2.0	<0.001	9.3 ± 3.7	6.5 ± 1.9	<0.001
Blood glucose level(mmol/L)	12.7 ± 4.0	9.6 ± 3.1	0.002	13.6 ± 4.1	10.0 ± 3.3	<0.001	12.7 ± 4.0	9.6 ± 3.1	<0.001
Serum ficolin-3 levels (μg/mL)	10.1 ± 4.1	16.9 ± 5.6	<0.001	11.4 ± 4.4	17.8 ± 5.5	<0.001	12.7 ± 4.5	19.0 ± 5.5	<0.001

Numerical variables were presented as mean ± standard deviation or median (interquartile range) and analyzed by unpaired Student’s *t* test or Mann–Whitney *U* test. Categorical variables were expressed as counts (percentage) and analyzed by chi-square test or Fisher’s exact test

*GCS* Glasgow Coma Scale, *CT* computerized tomography, *SAH* subarachnoid hemorrhage

Serum ficolin-3 levels statistically significantly predicted 1-week mortality of patients under ROC curve and a cutoff value of <13.3 μg/mL predicted the prognosis with sensitivity of 85.0 % and specificity of 76.9 %. Table 3 shows that the AUC of the serum ficolin-3 levels was similar to that of GCS scores for prediction of 1-week mortality of the patients. In a combined logistic-regression model, serum ficolin-3 levels improved the AUC of GCS scores, but the difference was not statistically significant.

**Table 3:** Analysis and comparison of area under receiver operating characteristic curve

Variables	1-week mortality prediction		6-month mortality prediction		6-month functional outcome prediction	
	AUC (95 % CI)	*P* value	AUC (95 % CI)	*P* value	AUC (95 % CI)	*P* value
GCS scores	0.883 (0.814–0.933)	Ref.	0.875 (0.805–0.927)	Ref.	0.872 (0.802–0.925)	Ref.
Ficolin-3 levels	0.836 (0.760–0.896)	0.305	0.818 (0.740–0.881)	0.186	0.815 (0.736–0.878)	0.196
GCS scores + ficolin-3 levels	0.915 (0.853–0.957)	0.294	0.904 (0.839–0.949)	0.232	0.900 (0.835–0.946)	0.207

A combined logistic-regression model was configured to estimate the additive benefit of ficolin-3 to GCS score. Comparisons of AUCs were performed using *z* test. GCS score + ficolin-3 level indicates the combined use of GCS score and ficolin-3 level to predict prognosis

*AUC* area under curve, *CI* confidence interval, *GCS* Glasgow Coma Scale, *Ref.* reference

### Prediction of 6-month mortality

In Table 2, serum ficolin-3 levels and other variables were highly associated with 6-month mortality of patients with STBI. A multivariate analysis demonstrated that GCS scores (OR 0.408, 95 % CI 0.271–0.613, *P* < 0.001) and serum ficolin-3 levels (OR 0.847, 95 % CI 0.756–0.950, *P* < 0.001) were the independent predictors for 6-month mortality of patients.

Serum ficolin-3 levels statistically significantly predicted 6-month mortality of patients under ROC curve, and a cutoff value of <13.4 μg/mL predicted the prognosis with sensitivity of 70.0 % and specificity of 83.0 %. Table 3 shows that the AUC of the serum ficolin-3 levels was similar to that of GCS scores for prediction of 6-month mortality of the patients. In a combined logistic-regression model, serum ficolin-3 levels improved the AUC of GCS scores, but the difference was not statistically significant.

### Prediction of 6-month unfavorable outcome

In Table 2, serum ficolin-3 levels and other variables were highly associated with 6-month unfavorable outcome of patients with STBI. A multivariate analysis demonstrated that GCS scores (OR 0.460, 95 % CI 0.336–0.632, *P* < 0.001) and serum ficolin-3 levels (OR 0.845, 95 % CI 0.758–0.942, *P* < 0.001) were the independent predictors for 6-month unfavorable outcome of patients.

Serum ficolin-3 levels statistically significantly predicted 6-month unfavorable outcome of patients under ROC curve, and a cutoff value of <18.0 μg/mL predicted the prognosis with sensitivity of 89.1 % and specificity of 60.9 %. Table 3 shows that the AUC of the serum ficolin-3 levels was similar to that of GCS scores for prediction of 6-month unfavorable outcome of the patients. In a combined logistic-regression model, serum ficolin-3 levels improved the AUC of GCS scores, but the difference was not statistically significant.

## Discussion

To our best knowledge, the current study, for the first time, investigated the serum ficolin-3 levels after STBI. Its main findings were that STBI patients had decreased serum ficolin-3 levels compared with healthy controls; serum ficolin-3 levels were independently associated with GCS scores and plasma C-reactive protein levels; and ficolin-3 was identified as an independent prognostic predictor and had high predictive value.

In agreement with previous reported data regarding acute ischemic stroke and aneurysmal subarachnoid hemorrhage [41, 42], the decreased serum ficolin-3 levels were found within 6 h after STBI in the current study. In ischemic stroke, the decreased serum concentration of ficolin-3 could be observed in the very early phase and remained unchanged during the next 3–4 days [41]. Since ficolin-3 has been shown to be involved in the sequestration of dying host cells [43], it seems reasonable to surmise that this decrease of serum ficolin-3 levels should be due to consumption through the binding of the molecules to the apoptotic and necrotic cells [44], indicating that the decreased levels in sera during the acute phase of head trauma could be related to the acute traumatic event.

In ischemic stroke, serum ficolin-3 levels are inversely correlated with the severity of stroke indicated by the National Institute of Health stroke scale on admission and the concentrations of S100b, an indicator of the size of cerebral infarct [41]. In aneurysmal subarachnoid hemorrhage, low levels of plasma ficolin-3 are related to hemorrhagic severity assessed using the World Federation of Neurosurgical Societies grading scale, vasospasm defined as neuro-worsening with angiographic confirmation of vessel narrowing and cerebral ischemia defined as hypodense lesion on CT scan performed before discharge [42]. This study demonstrated that the decreased serum ficolin-3 levels were highly associated with trauma severity reflected by GCS scores. Hence, the selective ability for complement activation after the binding of ficolin-3 to dying cells might be responsible for the selective clinical correlation with the levels of this protein [45], indicating serum fiction-3 levels could reflect the severity of brain injury.

The complement activation has been confirmed as a crucial inflammatory component in a lot of diseases [36, 37]. An increasing number of clinical researches have addressed the potential role of ficolin-3 in various inflammatory status and diseases like diabetic peripheral neuropathy [46], diabetic microvascular complication [47], and pre-eclampsia [48]. The current study found that serum ficolin-3 levels were associated negatively with systemic inflammatory severity indicated by plasma C-reactive protein levels. The decrease in ficolin-3 level has been confirmed to be accompanied with increased complement activation [49]. Thus, the increased consumption of ficolin-3 might exacerbate complement activation, leading to the inflammation and tissue damage after head trauma.

Lower serum ficolin-3 levels have been demonstrated to be highly associated with the severity and unfavorable outcome after acute ischemic stroke and aneurysmal subarachnoid hemorrhage [41, 42]. In this study, ficolin-3 was identified as an independent predictor for long-term and short-term clinical outcomes including 1-week mortality, 6-month mortality, and 6-month unfavorable outcome. We further used the ROC curves to verify the prognostic predictive values of serum ficolin-3 levels. Although ficolin-3 did not improve the AUC of GCS scores under ROC curve, ficolin-3 had similar predictive performance to the GCS scores. Thus, it is more convincing that ficolin-3 may be a good prognostic predictor after STBI.

## Conclusions

In this study, serum ficolin-3 levels are correlated with head trauma severity and systemic inflammatory severity, as well as independently predict short-term and long-term clinical outcomes of severe TBI. Therefore, it is suggested that serum ficolin-3 may have the potential to be a good prognostic predictive biomarker after head trauma.

## Abbreviations

AUC: area under curve
CI: confidence interval
CT: computerized tomography
GCS: Glasgow Coma Scale
MASP: MBL/ficolin-associated serine proteases
MBL: mannose-binding lectin
OR: odds ratio
ROC: receiver operating characteristic
SAH: subarachnoid hemorrhage
STBI: severe traumatic brain injury
